# Regulation of hormone pathways in wheat infested by *Blumeria graminis* f. sp. *tritici*

**DOI:** 10.1186/s12870-023-04569-1

**Published:** 2023-11-09

**Authors:** Shuangyu Bai, Jiaohui Long, Yuanyuan Cui, Zhaoyi Wang, Caixia Liu, Fenglou Liu, Zhangjun Wang, Qingfeng Li

**Affiliations:** School of Agriculture, Ningxia University, Yinchuan, 750021 China

**Keywords:** Wheat, *Blumeria graminis* f. sp. *tritici*, Hormone, Transcriptome, WGCNA

## Abstract

**Background:**

Wheat powdery mildew is an obligate biotrophic pathogen infecting wheat, which can pose a serious threat to wheat production. In this study, transcriptome sequencing was carried out on wheat leaves infected by *Blumeria graminis* f. sp. *tritici* from 0 h to 7 d.

**Results:**

KEGG and GO enrichment analysis revealed that the upstream biosynthetic pathways and downstream signal transduction pathways of salicylic acid, jasmonic acid, and ethylene were highly enriched at all infection periods. Trend analysis showed that the expressions of hormone-related genes were significantly expressed from 1 to 4 d, suggesting that 1 d-4 d is the main period in which hormones play a defensive role. During this period of time, the salicylic acid pathway was up-regulated, while the jasmonic acid and ethylene pathways were suppressed. Meanwhile, four key modules and 11 hub genes were identified, most of which were hormone related.

**Conclusion:**

This study improves the understanding of the dynamical responses of wheat to *Blumeria graminis* f. sp. *tritici* infestation at the transcriptional level and provides a reference for screening core genes regulated by hormones.

## Introduction

As an important staple crop, wheat is widely grown in many areas of the world, and its yield is of great significance in ensuring global food security [1]. Wheat powdery mildew caused by the obligate biotrophic fungal pathogen *Blumeria graminis* f. sp. *tritici* (*Bgt*) is one of the most detrimental wheat diseases and can cause serious economic losses in wheat production, possibly resulting in a yield loss of 10–40% [2]. In order to reduce the losses caused by *Bgt*, a lot of effort has been put into studying the genetic molecular mechanisms of wheat powdery mildew so that effective control measures can be developed [3–10].

There are fewer studies on powdery mildew and defence pathways in wheat and more on powdery mildew in other species [11–14]. It has been shown that salicylic acid (SA), ethylene (ET) and Brassinolide are involved in resistance to powdery mildew in cucumber [15]. SA is important in oxidative burst and systemic acquired resistance (SAR) after pathogen attack. Two SAR pathways exist in plants that can induce SAR, one regulated by SA and *NPR1* and the other by free radicals, such as nitric oxide and reactive oxygen species. SAR is stimulated to promote the accumulation of PR proteins to counteract secondary infection in neighboring plants [16]. Most of the signaling molecules involved in the induction of SAR can be found in the phloem at the time of infection [17]. *Bgt* can also promote infestation by regulating hormone biosynthesis [15]. Jasmonic acid (JA) signalling has an important role in wheat powdery mildew resistance, methyl jasmonate can activate *PR* gene expression during *Bgt* infestation [18], while powdery mildew can also activate JA synthesis and pathogenesis-related genes (PR) transcription [19]. Hormones have crosstalk mechanisms and have been extensively studied in plants. The SA signalling regulator enhanced disease resistance 1 (EDS1) interacts with DELLA proteins to regulate plant defence and growth relationships [20]. The SA receptor nonexpressor of PR genes 1 (NPR1) interacts with the gibberellin (GA) receptor GA-insensitive dwarf1 (GID1) to mediate crosstalk between SA and GA [21], and NPR1 also binds the ET signalling pathway transcription factor ethylene insensitive (EIN) to inhibit its activity [22]. Some WRKY transcription factors also have important roles in SA-JA crosstalk [23]. SA and JA can also affect each other's levels, with SA and methyl jasmonate (MeJA) treatments affecting the biosynthesis of JA and SA, respectively [24], reduced levels of JA increased plant susceptibility to *Botrytis cinerea* [25]. Plant hormones are important for plants in adaptation to abiotic stresses by mediating a wide range of adaptive responses which generally regulate the degradation of transcriptional regulators through the ubiquitin–proteasome system to rapidly alter gene expression [26].

In plant disease resistance studies, transcriptomes have been widely used to explore the interactions between host plants and pathogens, providing an important basis for the mining of plant disease resistance genes and the analysis of the corresponding disease resistance mechanisms [27, 28]. To date, a large number of transcriptomic studies on plant-pathogens have been reported, and the molecular mechanisms of resistance and regulation of gene expression have been discussed. For example, transcriptome analysis reveals important roles of phytohormones and the phenanthrene pathway in powdery mildew resistance in watermelon [29]. Gibberellin and abscisic acid are important in the treatment of *Fusarium head blight* in wheat through modulation of early plant hormone defense signals against *Fusarium graminearum* revealed through transcriptome and metabolome analysis [30]. Flavonoids were found to confer powdery mildew resistance in wheat by regulating the redox system through transcriptome and metabolome analysis [31]. The response of wheat to fungal infection is very complex, involving a series of biological responses and physiological processes.

To prevent infection by pathogens, plants have evolved a defense system controlled by plant hormones [32]. At present, the most studied defense pathways of plant disease resistance are SA, JA, and ET signal pathways [10, 33–35].

After being infected by pathogens, plants undergo hypersensitive responses (HR) at the infected site and SA is produced. SA, as a signal molecule, induces the expression of *PR* in infected tissues, by which the whole plant acquires disease resistance [36]. NPR1 has been shown to act as the receptor of SA [37, 38]. In the nucleus, *NPR1* regulated the expression of *PR* in response to SA, whereas in the cytoplasm *NPR1* plays a role in the antagonism of SA and JA [39]. JA, as a stress signaling molecule, is mainly involved in plant resistance to mechanical damage, chewing insects, and saprophytic pathogens [17, 40–42]. Upon damage induction, Jasmonat resistant 1 (JAR1) bind to JA to produce JA-Ile. JA-Ile is the active form of JA in Arabidopsis, which is perceived by coronatine-insensitive 1 (COI1). COI1, as an F-box protein [43], is involved in the formation of the E3 ubiquitin ligase complex of SCF (Skp/Cullin/F-box). JAZ can be ubiquitinated by SCF complex after damage induction and then degraded by 26S proteasome. Jasmonate ZIM domain protein (TIFY/JAZ) can bind and inhibit the transcriptional activity of jasmonate-insensitive 1(JAI1*/*MYC2). The degradation of JAZ can release the transcriptional activity of *MYCs,* which positively regulates the expression of JA-responsive genes [44, 45]. ET plays an important role in plant disease resistance as a defense hormone and is also involved in many abiotic stress responses [22, 23, 46–49]. ET receptors are very important in the ET signal transduction pathway [50]. In the presence of ET, the receptors will bind to ET and cannot bind to Constitutive Response 1 (CTR1) so that CTR1 cannot phosphorylate EIN2 [51]. The C-terminus of unphosphorylated EIN2 will be cleaved and transported to the nucleus to promote downstream signal transduction [52]. The transcription factors *EIN3/EILs* will receive the signal and bind to ethylene response factor 1 (*ERF1*) promoter to activate the downstream ET responses [53].

The aim of this study is to analyze the dynamic changes of hormone-related pathways in different stages of *Bgt* infection and provide insights into the defense responses of wheat to *Bgt* infection at the transcriptional level.

## Results

### Phenotypic observation of wheat infected by *Bgt*

**Fig. 1:**
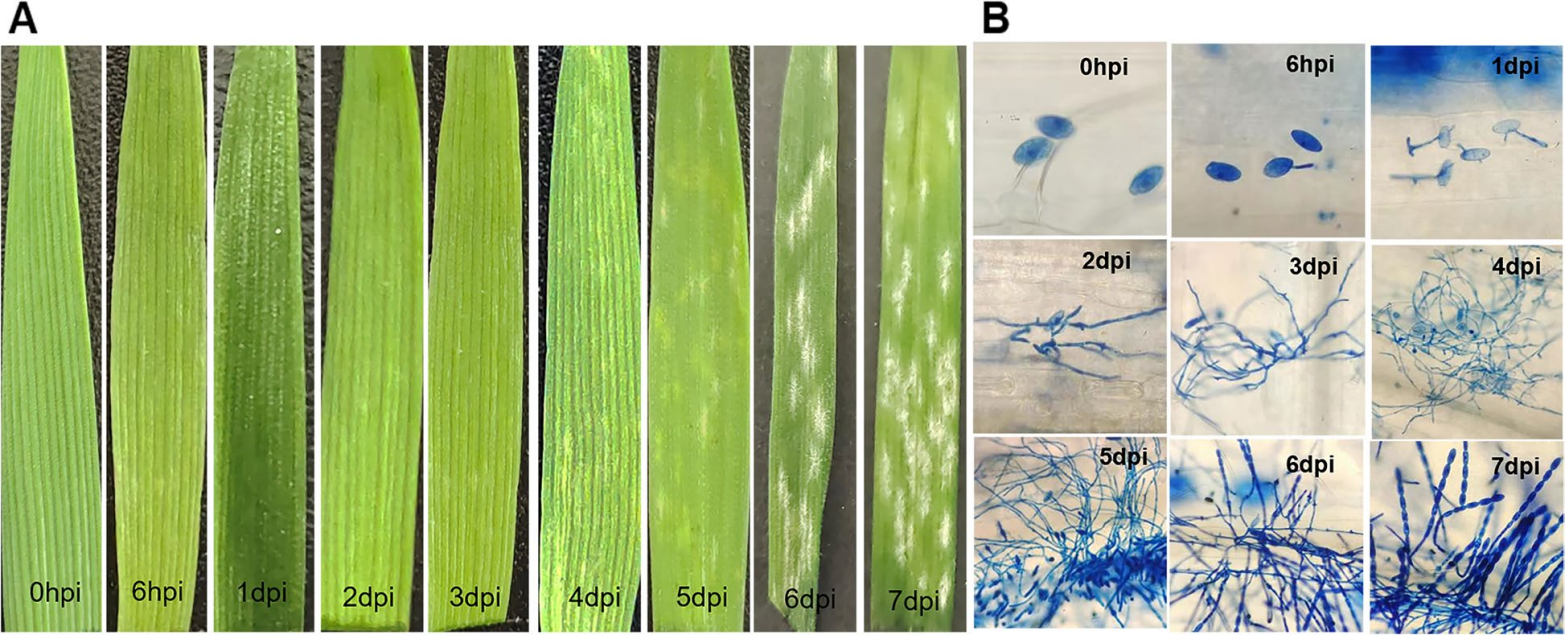
**A** Phenotypes of leaves at different time points after inoculation with powdery mildew. hpi: hours post inoculation, dpi: days post inoculation. **B** Asexual life cycle of *Bgt*

In order to clarify the dynamic responses of wheat leaves to *Bgt* infestation, *Bgt* inoculation was carried out at one leaf and one heart stage. To determine the time points for RNA-seq sampling, the responses of leaves to *Bgt* infestation and the growth of *Bgt* on leaves were observed at different time points. After 4 days post inoculation (dpi), mycelium appeared on leaves, and a large number of *Bgt* conidia were densely produced on leaves after 7 dpi (Fig. 1A). Trypan blue staining showed that appressorium formed after 1 dpi. Primary hyphae developed after 2 dpi. Conidia began to grow after 4 dpi. Bud tube developed at 6 dpi. Conidia and conidiophore were produced after 7 dpi, which became the center of competition for nutrients from wheat tissue cells (Fig. 1B). Based on the above results, 0 h, 6 h, 1 d, 4 d, and 7 d are the key points for the growth of *Bgt*.

### Identification of hormone-related differentially expressed genes

**Fig. 2:**
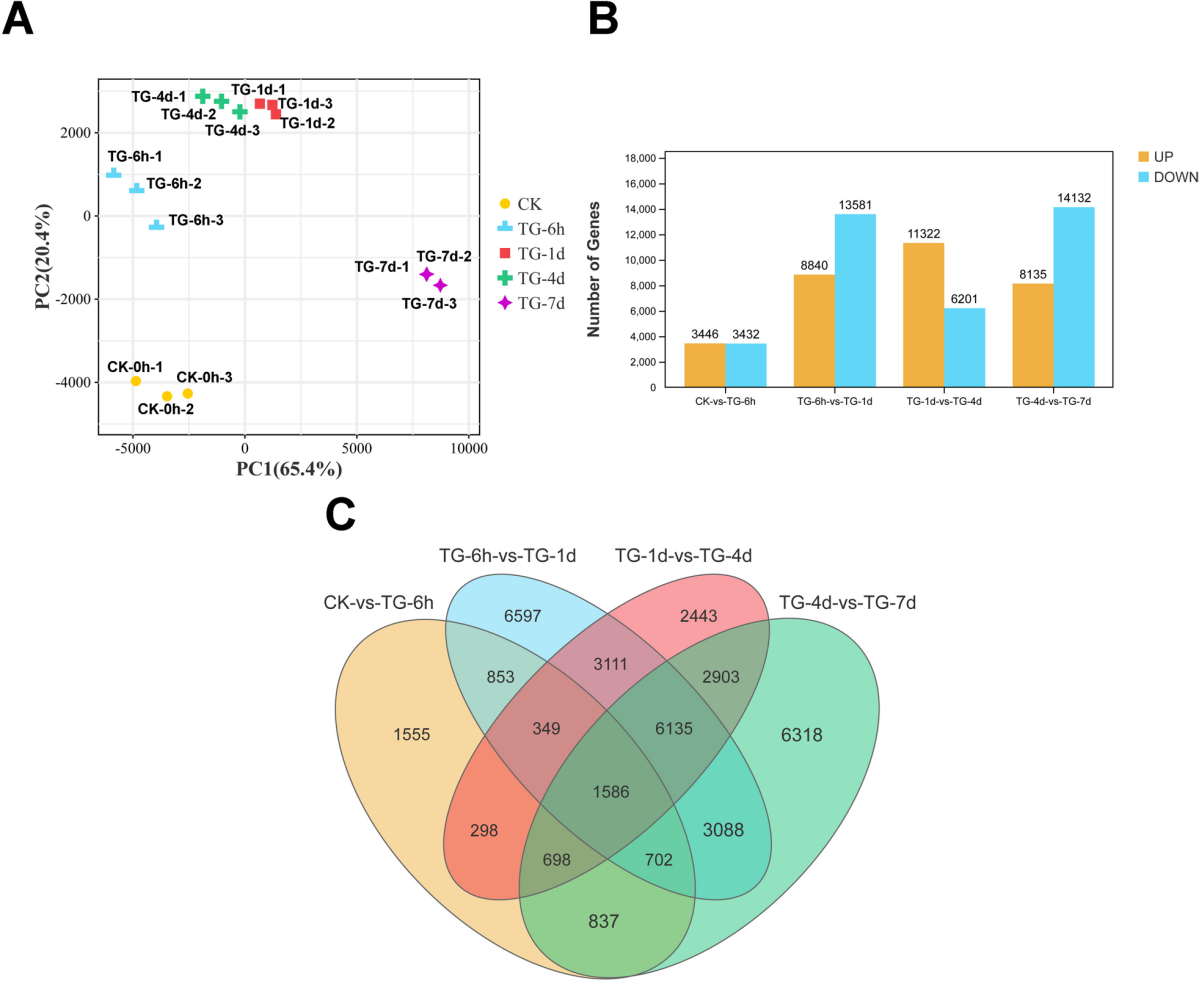
**A** Principal component analysis of samples. Clustering of samples at the same Inoculation time point. **B** Number of differently expressed genes at different infection time periods. **C** Venn Diagram of DEGs at different infection stages

A total of 120,711 genes were acquired from RNA seq data. In order to explore the plant-pathogen interactions during the whole process of powdery mildew infestation of wheat, samples from different infection stages were analyzed for differential gene expression. Principal Component Analysis (PCA) and correlation analysis between samples indicated that all three duplicates have high correlations, indicating the sequencing data is reliable (Fig. 2A). A total of 37,473 DEGs were identified in wheat leaves at different time points of *Bgt* infection. Among them, 6878 (3446 upregulated and 3432 downregulated), 22,421 (8840 upregulated and 13,581 downregulated), 17,532 (11,322 upregulated and 6201 downregulated), and 22,267 (8135 upregulated and 14,132 downregulated) DEGs were identified at 0 h-6 h, 6 h-1 d, 1 d-4 d, and 4 d-7 d, respectively (Fig. 2B). There were 1586 genes that responded at all periods of infestation (Fig. 2C).

**Fig. 3:**
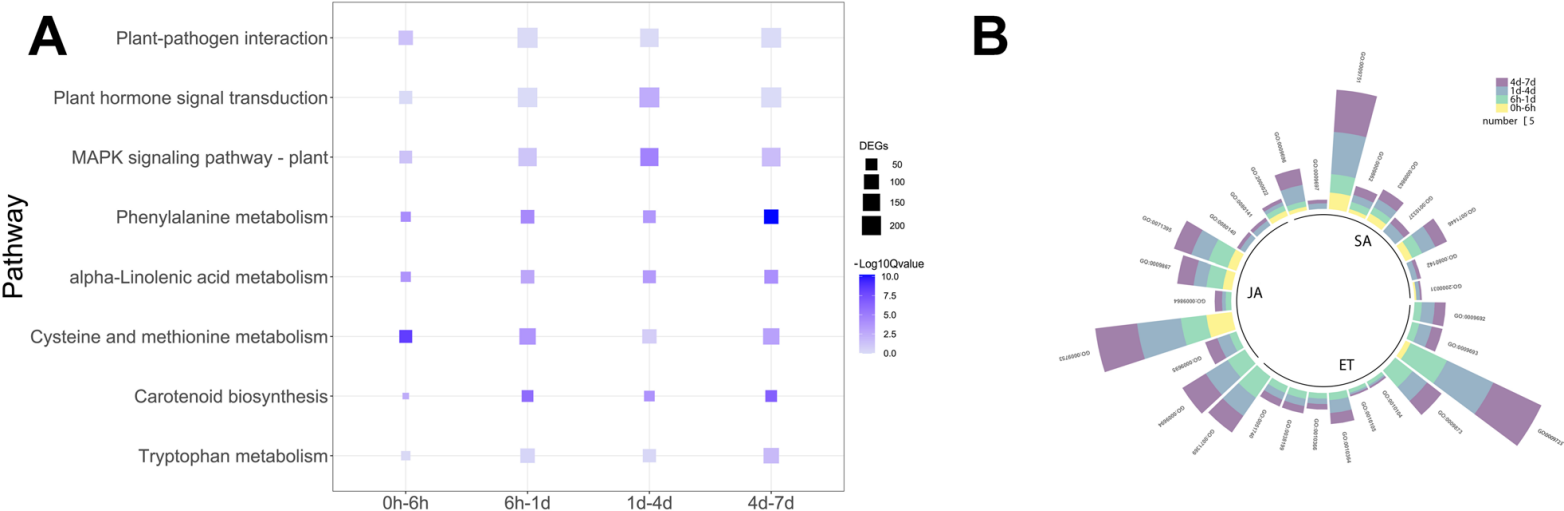
**A** Pathways of interest after KEGG enrichment analysis of differentially expressed genes. **B** Pathways of interest after GO enrichment analysis of differentially expressed genes. Copyright permission has been granted for related KEGG images

To determine the involvement of hormone-related metabolic pathways at different stages, DEGs were enriched at each stage using KEGG. Plant-pathogen interaction, plant hormone signal transduction, and MAPK signaling pathways were highly enriched, among which plant-pathogen interaction and plant hormone signal transduction were significantly enriched only at 0 h-6 h and 1 d-4 d, respectively, while MAPK signaling pathway was significantly enriched except at 6 h-1 d. There are biosynthetic pathways upstream and signal transduction pathways downstream of hormones. Phenylalanine metabolism related to SA biosynthesis, carotenoid biosynthesis related to Abscisic acid biosynthesis and α-linolenic acid metabolism related to JA biosynthesis were significantly enriched in all infection periods, while cysteine and methionine metabolism related to ET biosynthesis was significantly enriched in periods other than 1 d-4 d and tryptophan metabolism related to auxin biosynthesis was significantly enriched only at 4 d-7 d (Fig. 3A). Above results suggested that hormones play important roles in resistance to *Bgt* infestation.

Nine SA-related pathways, nine JA-related pathways, and eleven ET-related pathways were enriched by GO analysis, among which response to salicylic acid (GO: 0009751), response to jasmonic acid (GO: 0009753) and response to ethylene (GO: 0009723) have the highest number of DEGs. The response to salicylic acid pathway (GO: 0009751) was significantly enriched at 1 d-4 d and 4 d-7 d, while the response to jasmonic acid pathway (GO: 0009753) were significantly enriched at 0 h-6 h and 1 d-4 d. In 0 h-6 h period, 6 SA-related pathways and 4 JA-related pathways were enriched, but only one ethylene related pathway (GO: 0009723) was enriched (Fig. 3B). After 6 h, the number of DEGs in ET-related pathway was significantly increased. Above results imply that SA-, JA-, and ET-related pathways are important in the response of wheat to pathogens, with SA- and JA-related pathways function at all the stages and ET-related pathway may function mainly at late stages of infestation.

### Trend analysis reveals specific expression patterns of wheat resistance to *Bgt*

**Fig. 4:**
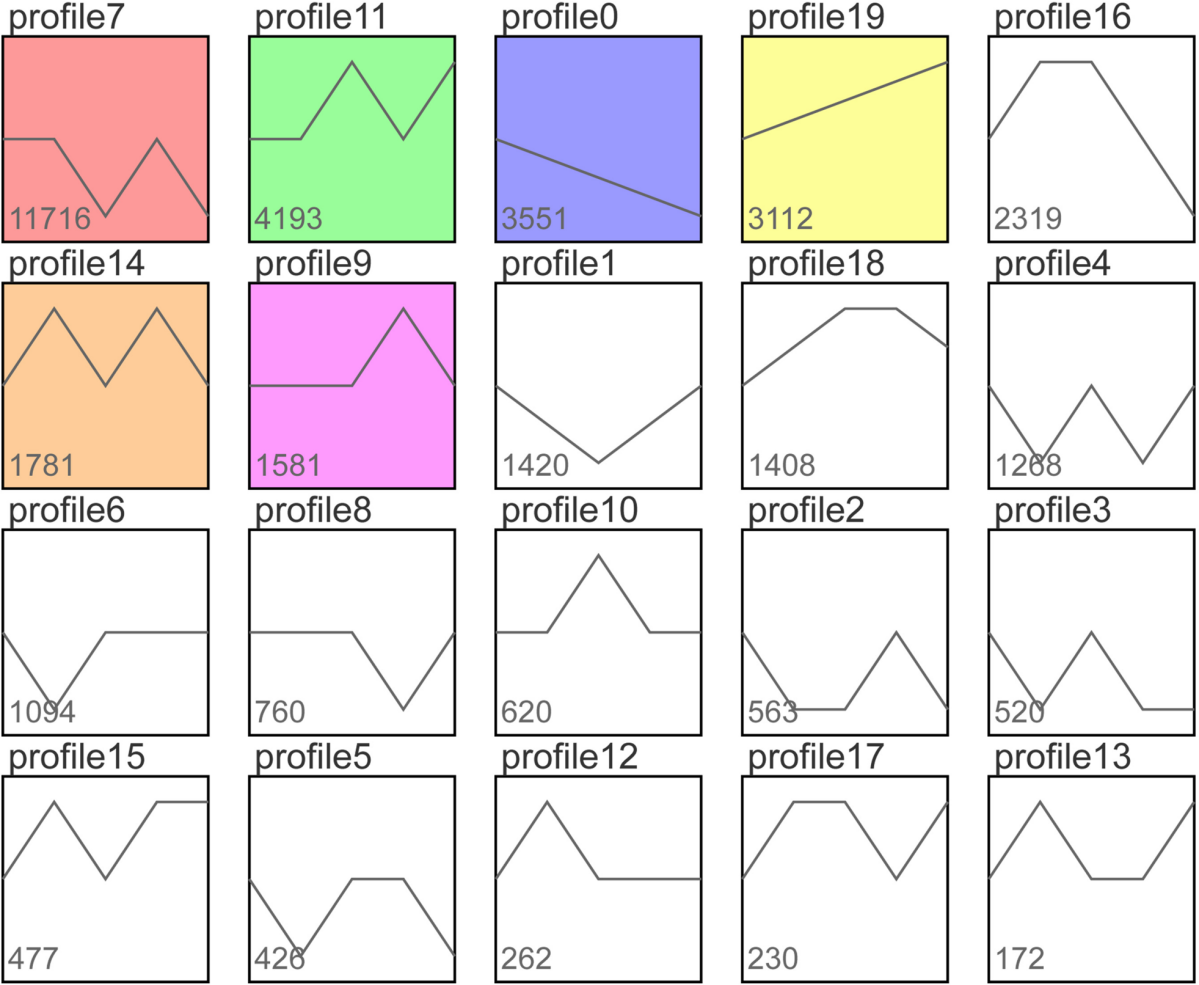
Trend plots, with colored trend plots showing significant gene concentration (*P* < 0.05)

Trend analysis of 37,473 DEGs enriched six significant trends, namely profile0, profile7, profile9, profile11, profile14 and profile19 (Fig. 4).

**Fig. 5:**
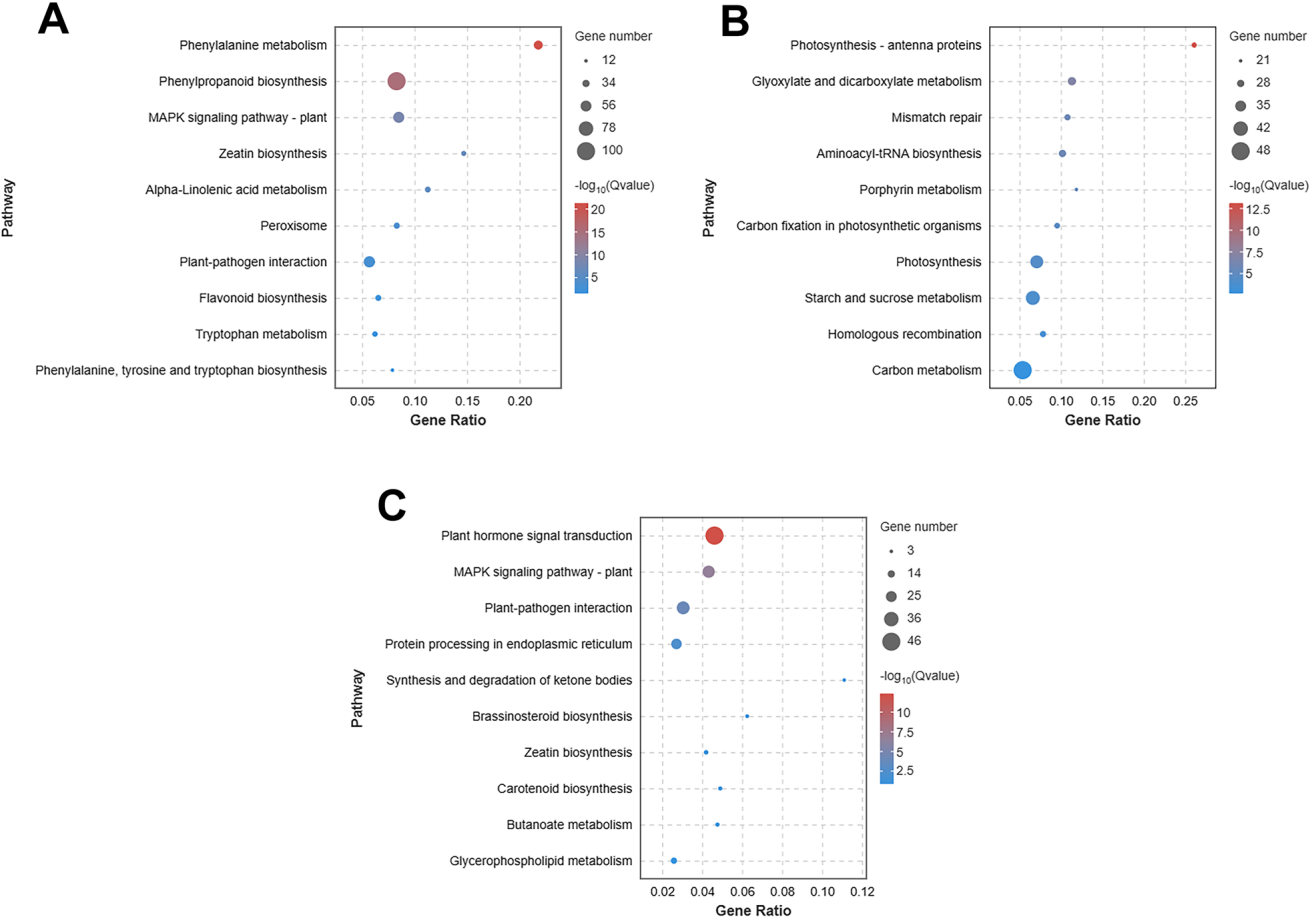
**A** Significant bubble plot for profile19. **B** Significant bubble plot for profile0. **C** Significant bubble plot for profile9

Genes in profile19 were consistently upregulated during the infestation. Profile19 contained 3112 DEGs involved in salicylic acid, jasmonic acid, cytokinin, and auxin-related phenylalanine metabolism, α-linolenic acid metabolism, zeatin biosynthesis, and tryptophan metabolism pathways. Notably, the MAPK signaling pathway, phenylpropane metabolism, plant-pathogen interaction, and flavonoid biosynthesis were significantly enriched in profile19 (Fig. 5A).

Genes in profile0 were consistently downregulated throughout the infestation period. Profile0 contained 3551 DEGs, mainly enriched in photosynthesis-antenna protein, glyoxylate and dicarboxylate metabolism, mismatch repair, aminoyl-tRNA biosynthesis, carbon fixation in photosynthetic organisms, porphyrin metabolism, starch and sucrose metabolism, photosynthesis, homologous recombination and carbon metabolism pathways (Fig. 5B), suggesting that *Bgt* may create favorable environment for its growth by affecting genes in these pathways.

Profile9 contains a total of 1581 genes. These genes are mainly enriched in plant hormone signal transduction, plant-pathogen interaction, protein processing in the endoplasmic reticulum, and MAPK signaling pathway-plant pathways (Fig. 5C). The expression of these genes was consistent in 0 h-6 h and 6 h-1 d, but increased in 1 d-4 d, and then decreased in 4 d-7 d.

### Analysis of differentially expressed genes related to hormone signaling pathways in wheat

**Fig. 6:**
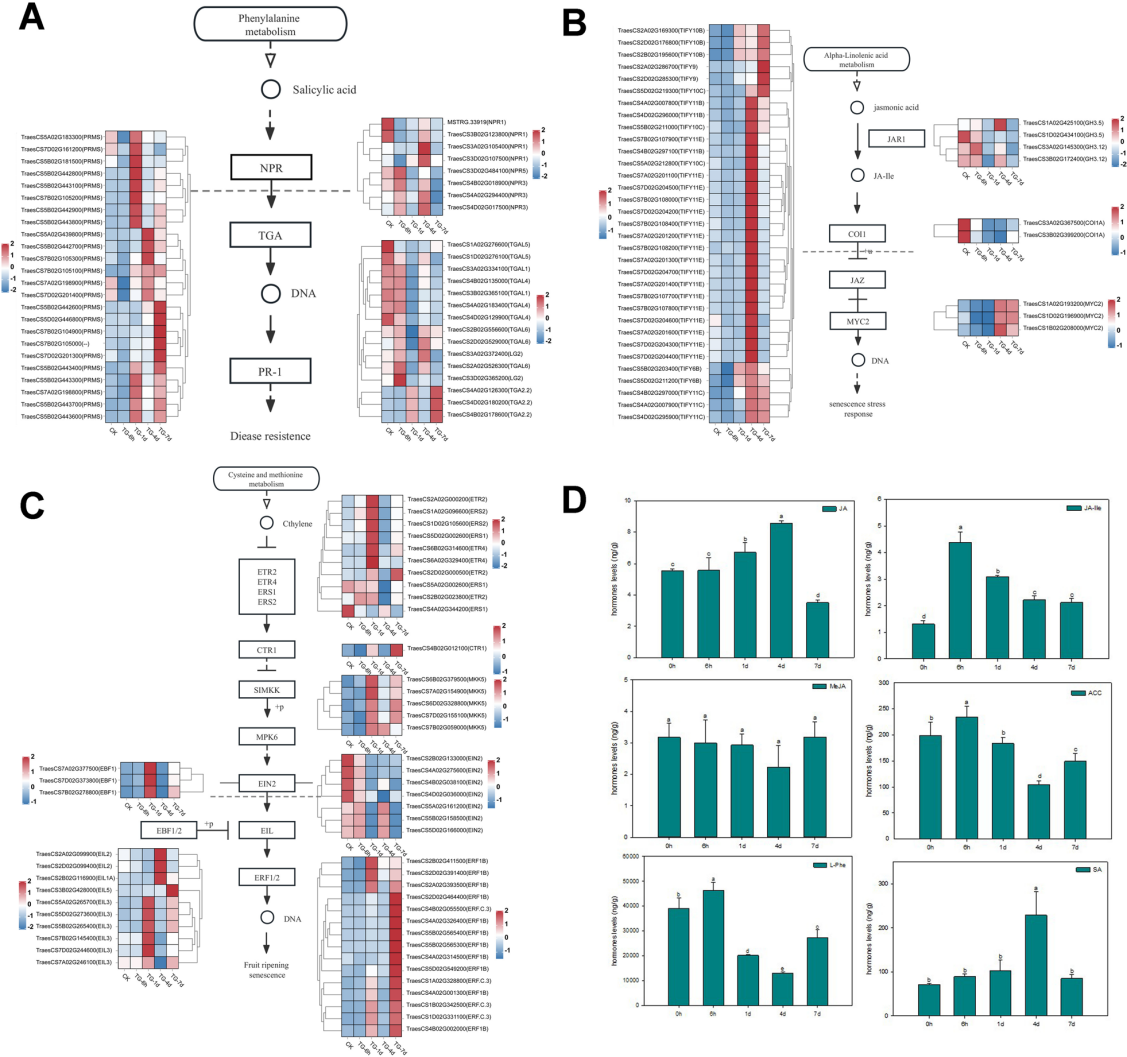
**A** Analysis of salicylic acid signaling pathway and its differential gene expression. **B** Analysis of jasmonic acid signaling pathway and its differential gene expression. **C** Analysis of ethylene signaling pathway and its differential gene expression. The FPKM values of the differential genes were normalized, and the heat map data was the average of the FPKM values of three repeats. Copyright permission has been granted for related KEGG images. **D** Analysis of hormone levels in wheat leaves after *Bgt* infestation. Three biological replicates were performed for each experiment. Bars with different letters indicate significant difference between treatments according to Duncan’s multiple range test, *p* < 0.05

In this study, we analyzed the expression patterns of DEGs related to hormone signaling pathways such as SA, JA, and ET signal pathways. For the SA signaling pathway, the expression of *NPRs*, *TGA*, and *PR* were analyzed. The expression of *NPR1* was high at 0 h (CK) and decreased at 6 h, while the expression of *NPR3,* which mediates the degradation of E3 ubiquitination ligase dependent NPR1 [54], was opposite to that of *NPR1*, with a low expression at 0 h and a relative high after 6 h. Interestingly, the expression of *NPR1*, *NPR5,* and *NPR3* were high at 4 d and decreased at 7 d. Most *TGA* transcription factors had high expressions at 0 h and 6 h, except for three transcripts annotated as *TGA2.2*. Most of the *PR* genes had high expressions at 7 d. Overall, *NPR1* and *TGA* were highly expressed at 0 h, 6 h, and 4 d, whereas *PR* was highly expressed at 7 d (Fig. 6A). Most *NPR*, *TGA*, and *PR* genes had low expressions at 1 d, with only a few *PR* genes being relatively highly expressed.

For the JA signaling pathway, the expression pattern of *JAR1*, *COI1*, *JAZ*, and *MYC2* were analyzed (Fig. 6B). JA-Ile, synthetized by JAR1, can catalyze the interaction of COI1 dependent SCF^COI1^ ubiquitin ligase complex with JAZ, and the degradation of JAZ by 26S proteasome [43]. The degradation of JAZ will release MYC2 and induce the expression of resistance genes. Our results showed the expressions of *JAR1* and *COI1* were high at 0 h, while the expression of *JAZ* and *MYC2* were relatively low at 0 h and 6 h. In addition, the expression of *JAZ* was highest at 4 d and decreased at 7 d, while the expression of *MYC2* remained consistent at 4 d and 7 d.

ET receptors and CTR1 negatively regulate the ET signaling pathway. Most of the ET receptors, for example, ethylene response sensor 1 (ERS1), ERS2, ethylene receptor 2 (ETR2), and *ETR4* reached the highest expression at 1 d, while *CTR1*, which binds to the ET receptors, had high expressions at 1 d and 7 d. In contrast, the positive ET regulatory factor *EIN2* located downstream of *CTR1* had low expressions on 1 d and 7 d, and high expressions at 0 h, 6 h, and 4 d. Low expression of *CTR1* can reduce the phosphorylation of EIN2 and release CEND. CEND can inhibit the expression of *EBF1/2* after entering the nucleus, while enhance the expression of downstream transcription factor *EIL*. EBF1/2 can bind EIL and degrade it by the 26S proteasome [36, 55–57]. *EBF1/2* showed low expressions at 0 h, 6 h, and 4 d, and *EIL* had high expressions at 1 d and 7 d. EIL can promote the transcription of *ERF1/2,* which will further promote the regulation of downstream resistance genes. Most of the *ERF1/2* genes exhibited high expressions at 7 d (Fig. 6C).

Hormone levels were examined by liquid chromatography tandem mass spectrometry (LC–MS/MS). From 1 to 4d, the decrease level of L-Phenylalanine, a precursor of SA, and the increased level of SA implied an up-regulation of the SA signaling pathway, while the decrease levels of JA-Ile and ET precursor 1-Aminocyclopropanecarboxylic acid (ACC) implied down-regulations of the JA and ET signaling pathways (Fig. 6D). The rise in both JA and SA concentrations indicated that there was less likely a completely antagonism but rather an intricate and well-regulated crosstalk between SA and JA during wheat response to pathogen.

### Weighted gene co-expression network analysis to screen for hub genes

**Fig. 7:**
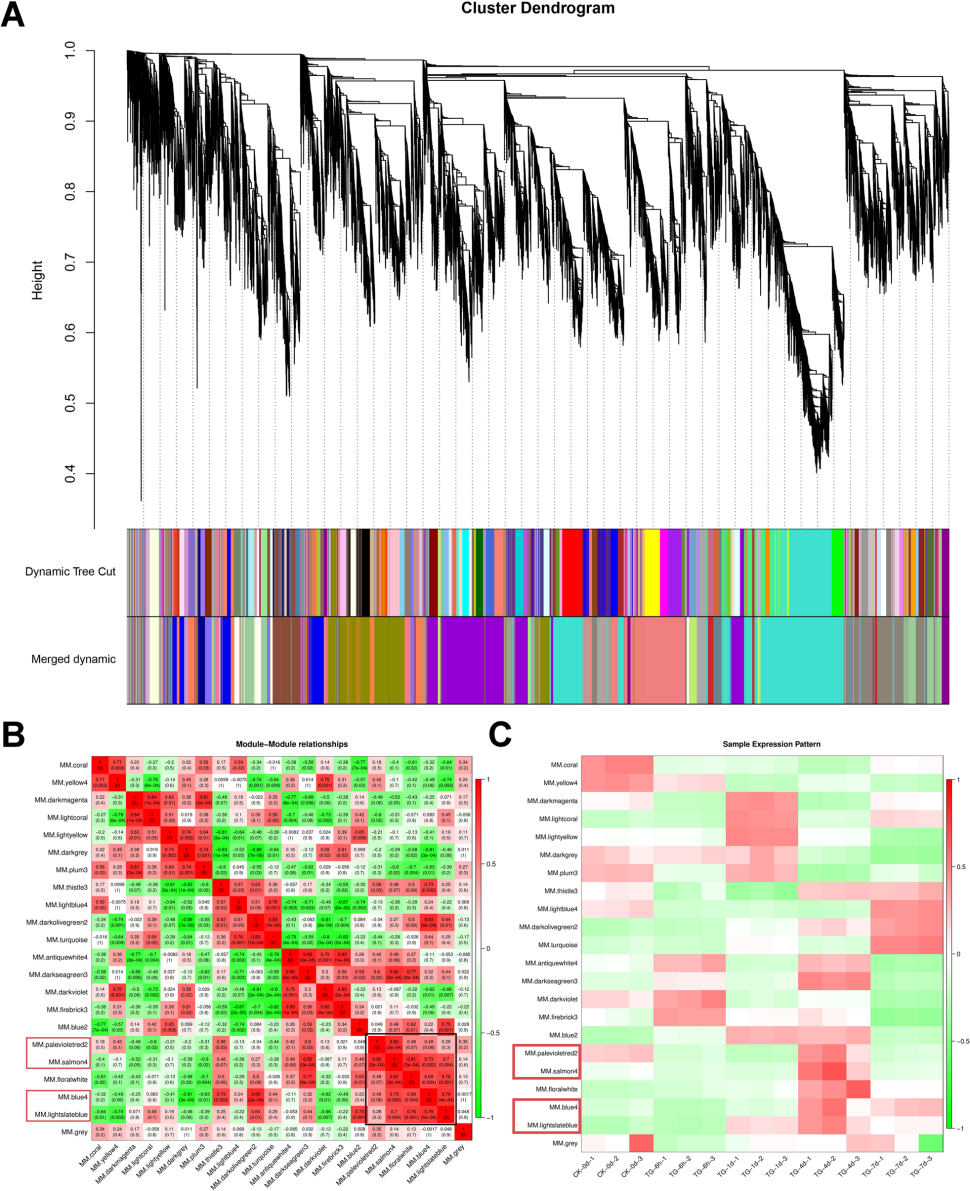
**A** Module hierarchy clustering plot. Each color represents a module, while gray represents genes that cannot be classified into any module. **B** Correlation heat map between modules. The numbers in the block represent the Pearson correlation coefficients of the two modules. **C** Sample expression pattern analysis plot. The horizontal axis represents 15 samples, and the vertical axis represents modules

To identify the gene regulatory network of wheat to *Bgt* infection, Weighted Gene Co-expression Network (WGCNA) was performed on 24,209 genes with a soft threshold of 16. Genes were clustered into preliminary modules initially, which were then merged by similarity into 22 final modules containing 113–4896 genes (Fig. 7A). Modules MM. blue4, MM. lightslateblue, MM. palevioletred2, and MM. salomn4, containing 217, 349, 113, and 971 genes, respectively, were highly correlated (Fig. 7B, C). Among them, the correlation coefficients of MM. salomn4 module and the other three modules were all greater than 0.7, indicating that MM. salomn4 module may be the key module.

**Fig. 8:**
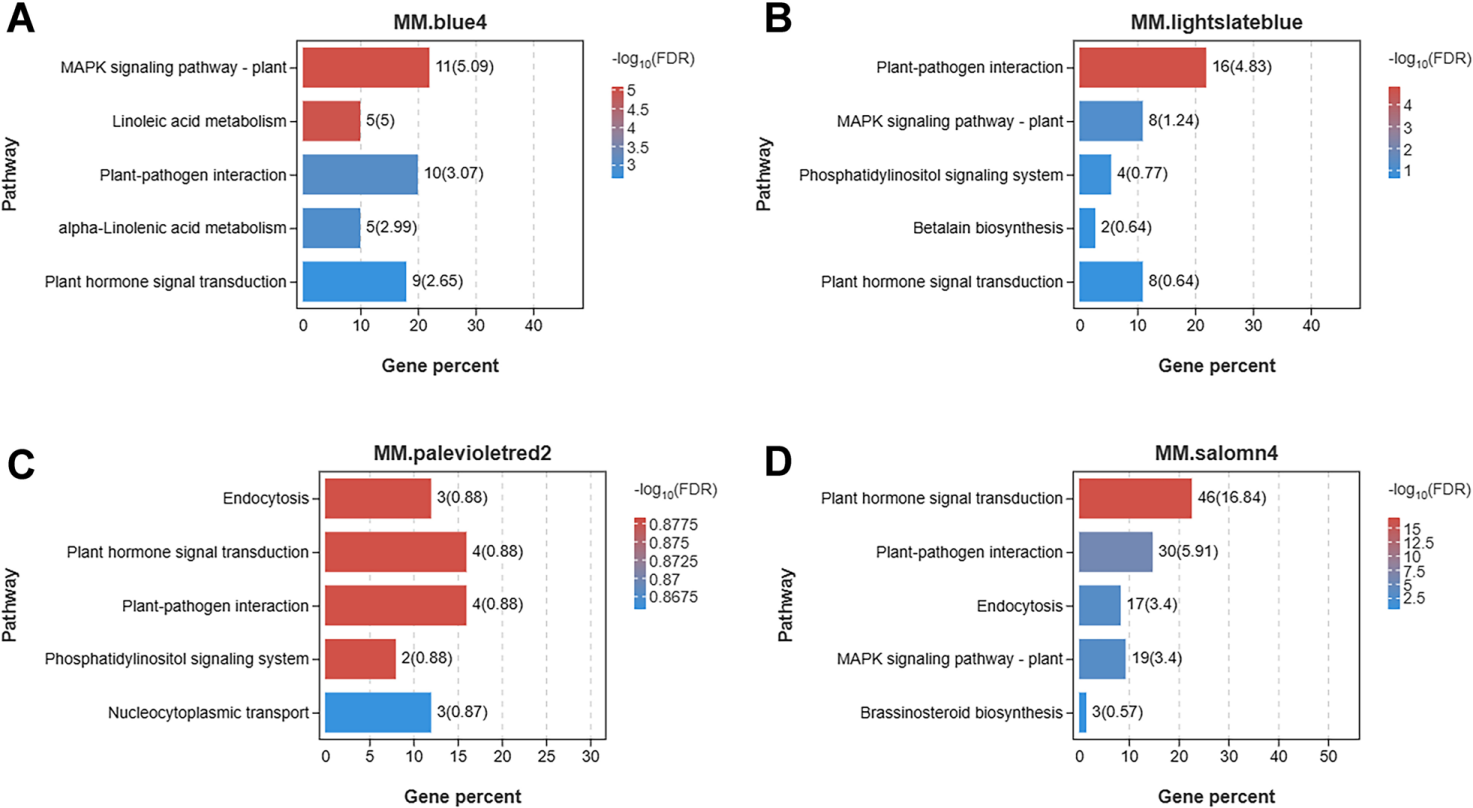
Four focus modules KEGG enrichment bar plot. **A** MM. blue4 module. **B** MM. lightslateblue module. **C** MM. palevioletred2 module. **D** MM. salomn4 module. The first five pathways enriched in these four modules, with adjusted *p* values (Q value, FDR) < 0.05, indicate significant enrichment of this pathway. Copyright permission has been granted for related KEGG images

**Fig. 9:**
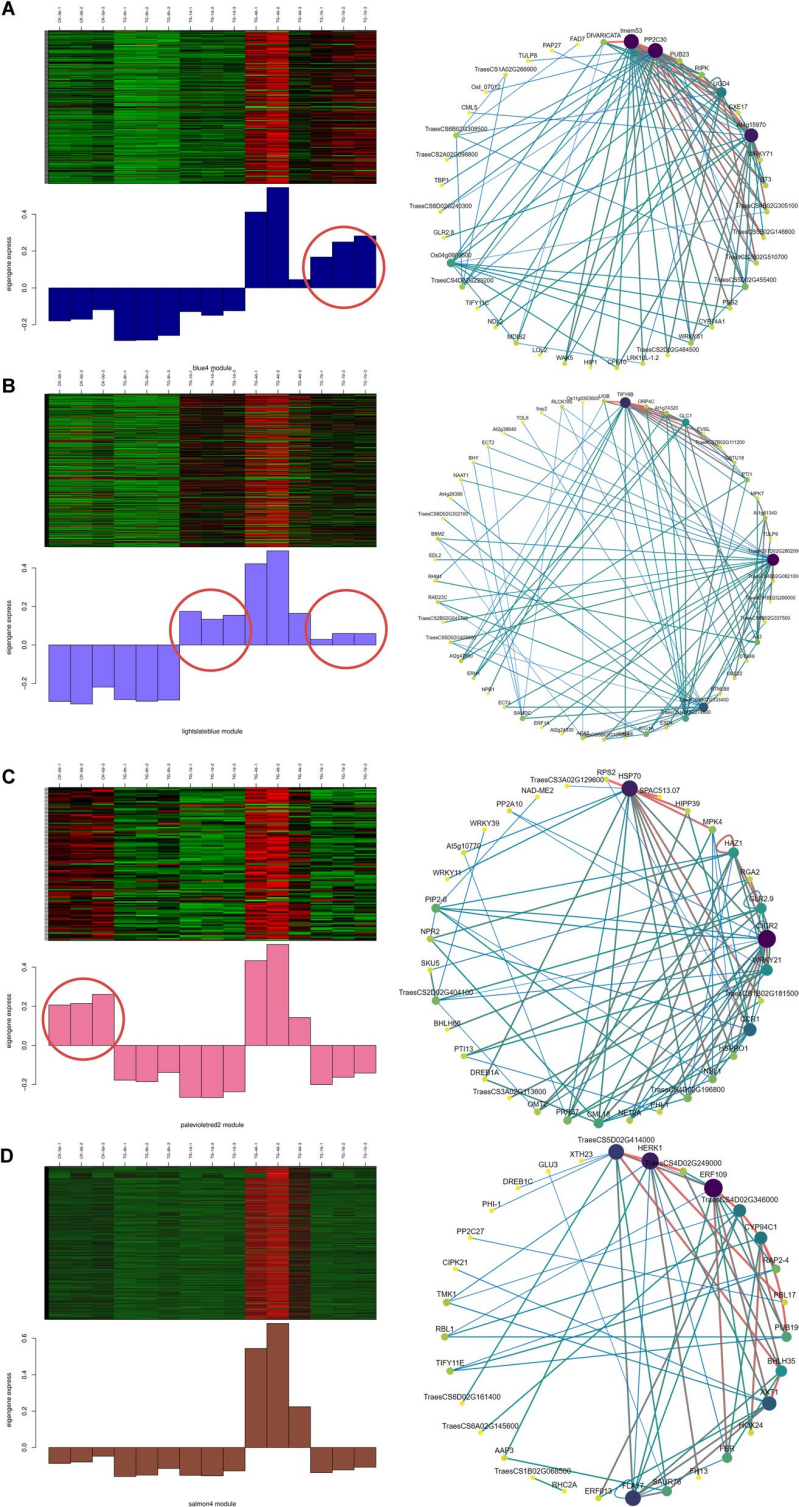
Gene distribution maps within the four module samples. Four modules were identified during different stages of *Bgt* infection. **A** MM. blue4 module. **B** MM. lightslateblue module. **C** MM. palevioletered2 module. **D** MM. salomn4 module. The heat map shows the co-expressed genes in each module, and on the right is the co-expressed network constructed by the top 100 pairs of genes with strong connectivity

These four modules were characterized according to KEGG enrichment. MM.blue4 module was significantly enriched for MAPK signaling, linoleic acid metabolism, plant-pathogen interaction, α-linoleic acid metabolism, and plant hormone signal transduction pathways (Fig. 8A). MM. lightslatebule module significantly enriched plant-pathogen interaction pathway (Fig. 8B). The MM. palevioletred2 module, although did not significantly enrich any pathway (Fig. 8C), contained four genes each in the phytohormone signaling and plant-pathogen interaction. The MM. salomn4 module was significantly enriched to the phytohormone signaling, plant-pathogen interaction, endocytosis, and MAPK signaling pathways (Fig. 8D). The expression patterns of the four modules were similar in that their module eigenvalues, all relatively low at 6 h and relatively high at 4 d. In addition, the expressions of these four modules were highest at 4 d among all the sampled periods. Differently, the comprehensive expression of MM. salomn4 was greater than 0 only at 4 d, while the comprehensive expressions of MM. blue4, MM. lightslateblue, and MM. palevioletered2 were greater than 0, respectively, at 4 d and 7 d, 1 d, 4 d and 7 d, and at 0 h and 4 d (Fig. 9A, B, C, D).

A total of 11 hub genes were identified by mapping the gene regulatory network based on the top 100 linkage pairs. Among them, three genes *TraesCS7B02G502200* (*tmem53*), *TraesCS4B02G210100* (*PP2C30*), and *TraesCS5D02G536500* (*At4g15970*), two genes *TraesCS5A02G204900* (*TIFY6B*) and *TraesCS1D02G280200,* two genes *TraesCS4B02G205700* (*HSP70*) and *TraesCS2A02G189600* (*CIGR2*), and four genes *TraesCS5D02G414000*, *TraesCS7D02G338800* (*HERK1*), *TraesCS1A02G370700* (*ERF109*), and *TraesCS2B02G263900* (*FLA17*) were identified in the module MM. blue4, MM. lightslateblue, MM. palevioletred2, and MM. salomn4 modules, respectively.

The function of these genes was predicted by NCBI-CDD, blastn and gene symbol numbers. The hub gene-related information is shown in Table 1. *TraesCS7B02G502200* (*tmem53*) is a transmembrane protein containing the DUF829 domain. *TraesCS4B02G210100* (*PP2C30*) is a protein phosphatase 2C mainly involved in plant hormone signal transduction and can serve as a key regulator of the abscisic acid signaling pathway [58]. *TraesCS5D02G536500* (*At4g15970*) is a member of the glycosyltransferase family. *TraesCS5A02G204900* (*TIFY6B*) is annotated as a transcription inhibitor containing the ZIM domain of JA, also known as *JAZ*, which negatively regulates the JA signaling pathway [59]. *TraesCS1D02G280200* has no structural domains predicted by NCBI-CDD and has two transcript variants. *TraesCS4B02G205700* (*HSP70*) encodes a heat shock protein. Numerous studies have shown a correlation between plant pathogen resistance and HSP70 [60, 61]. *TraesCS2A02G189600* (*CIGR2*) contains the GRAS domain, which is annotated as a chitin induced gibberellin response gene and mediates hypersensitive cell death during pathogen infection [62]. *TraesCS5D02G414000* contains the DUF4228 domain and is an uncharacterized gene. *TraesCS7D02G338800* (*HERK1*) with four transcript variants encodes a receptor like protein kinase, which is a single transmembrane protein located on the cell membrane, including an extracellular receptor domain that senses external signals, a transmembrane domain, and an intracellular kinase domain [63]. *TraesCS1A02G370700* (*ERF109*) was predicted to be an ET responsive transcription factor. *ERF109* regulates leaf purpose immunity, photosynthesis, and iron homeostasis and also mediates crosstalk between JA and growth hormone biosynthesis during lateral root formation in *Arabidopsis* [64, 65]. *TraesCS2B02G263900* (*FLA17*) encodes a fasciclin-like arabinogalactan protein, which is mostly studied in the aspects related to cell development. Most of the above mentioned hub genes are disease resistance related genes, which provide suggestions for the study of plant disease resistance. For the 11 hub genes, further research through emerging means (gene editing, gene silencing, etc.) may be needed.

**Table 1:** The hub gene identified by WGCNA

Gene ID	Gene symbol	Description	modules
TraesCS7B02G502200.1	tmem53	VAI94194.1 unnamed protein product	MM. blue4
TraesCS4B02G210100.1	PP2C30	Serine/threonine protein phosphatase 2C 30	MM. blue4
TraesCS5D02G536500.1	At4g15970	XP_020198537.1 uncharacterized protein At4g15970-like	MM. blue4
TraesCS5A02G204900.1	TIFY6B	QBQ83003.1 jasmonate ZIM-domain transcriptional repressor	MM. lightslateblue
TraesCS1D02G280200.2	~ ~	XP_020184869.1 uncharacterized protein LOC109770573 isoform X3	MM. lightslateblue
TraesCS4B02G205700.1	HSP70	VAI06780.1 unnamed protein product	MM. palevioletred2
TraesCS2A02G189600.1	CIGR2	VAH28547.1 unnamed protein product	MM. palevioletred2
TraesCS5D02G414000.1	~ ~	VAI22368.1 unnamed protein product	MM. salomn4
TraesCS7D02G338800.3	HERK1	XP_020176449.1 receptor-like protein kinase HERK 1 isoform X1	MM. salomn4
TraesCS1A02G370700.1	ERF109	VAH11080.1 unnamed protein product	MM. salomn4
TraesCS2B02G263900.1	FLA17	VAH46043.1 unnamed protein product	MM. salomn4

### Quantitative real-time PCR analysis

**Fig. 10:**
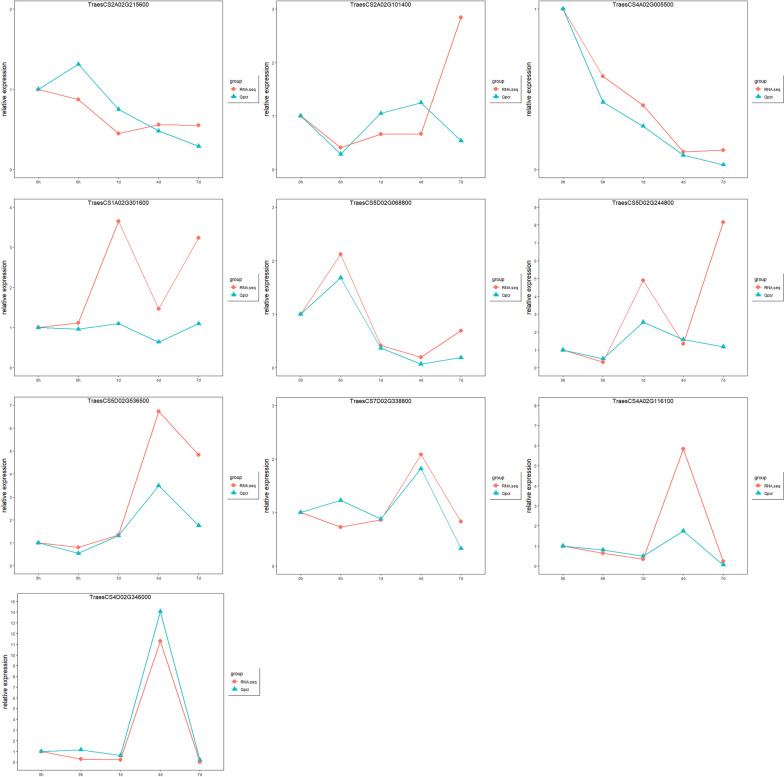
The qRT-PCR validation of disease-resistance-associated DEG. Five periods of infestation with three replicates of each period were used for qRT-PCR, and the validation results showed similar expression patterns to the RNA-seq results

To verify the reliability of the RNA-seq data, ten DEGs were selected for qRT-PCR. The results showed that the expression patterns of these genes at different periods of infestation were generally consistent with the results of RNA sequencing data (Fig. 10).

## Discussion

In this study, five key time points for *Bgt* growth were identified by observing the growth status of *Bgt* in leaves. Meanwhile, the transcriptomes of wheat leaves inoculated at 0 h, 6 h, 1 d, 4 d, and 7 d were sequenced and analyzed for DEGs enrichment in the four infestation periods (0 h-6 h, 6 h-1 d, 1 d-4 d, 4 d-7 d). These four infection stages were classified as initial stage, early stage, middle stage, and late stage. Studies have shown that different hormones change differently in wheat leaves at different infestation periods.

A large number of DEGs related to hormone pathways were identified by KEGG and GO enrichment analyses. Among them, four hormones, SA, JA, ET, and ababolic acid, played important roles in wheat response to *Bgt* infection. These hormones exert their functions at mid-infestation (1 d-4 d), where most of the genes in the SA pathway are upregulated, while most of the genes in the JA and ET pathways are downregulated. In addition, the plant-pathogen interaction pathway was significantly enriched only at the initial stage of infection (0 h-6 h). MAPK signaling pathway was significantly enriched at the initial, middle, and late stages of infection (0 h-6 h, 1 d-4 d, and 4 d-7 d). Receptor-like kinase (RLK) and receptor-like protein (RLP) play crucial roles in plant pathogen recognition by recognizing pathogen-associated molecular patterns (PAMP), inducing mitogen-activated protein kinase (MAPK) phosphorylation cascade reactions to induce the expression of immune genes [66, 67]. Genetic and molecular analyses have shown extensive crosstalk between SA and JA/ET mediated defense signaling pathways in a synergistic or antagonistic manner [68]. SA-JA crosstalk is not always antagonistic but varies according to conditions. For example, the expressions of SA and JA responsive genes were synergistic at low concentrations of SA and JA, while antagonistic effects were observed at high concentrations of SA and JA [69]. Antagonism between SA and JA usually occurs through regulating protein NPR1, which is related to systemic acquired resistance and induced systemic resistance [70]. Trend analysis revealed that the continuously upregulated DEGs mainly enriched in the biosynthetic pathways upstream of hormones, including phenylpropanoid metabolism, flavonoid metabolism, and plant-pathogen interaction. This indicated the important roles of these pathways throughout the entire infection period. Phenylpropane metabolism provides plants with flavonoids and lignans for cell wall reinforcement. Phytoalexins synthesis increased long-distance transport of water and nutrients and protected plants against mechanical invasion by pathogens [71, 72]. Flavonoids are low molecular weight antioxidants that are involved in a variety of compositions as defense compounds, such as UV radiation resistance, anthocyanins, and phytoalexins synthesis [73, 74]. The increase in Methyl salicylate content can stimulate the phenylpropanoid pathway, resulting in an increase in leaf flavonoid content [75]. However, some genes involved in pathways such as photosynthesis, starch and sucrose metabolism, and carbon metabolism exhibited continuous down-regulation, indicating that plant growth and carbohydrate accumulation were affected throughout the entire infection period. Gao et al. found that carbon metabolism was mainly regulated by the redox state of cells, and the starch content decreased after *Bgt* infection [76]. Genes involved in the downstream pathways of hormones were upregulated at the middle stage of infection (1 d-4 d) and then downregulated at the late stage (4 d-7 d). The upstream biosynthetic pathway of hormones is continuously upregulated, while the downstream signal transduction pathway is upregulated after 1 d and downregulated after 4 d, by this time *Bgt* had fully colonised the plant cells and presumably the relevant intracellular regulatory pathways were disturbed.

The expression analysis of DEGs in SA, JA, and ET signaling pathways showed that the *PR* genes of the SA signaling pathway were expressed at 1 d, 4 d, and 7 d. The core transcription factors *MYC* and *ERF* of JA and ET pathway showed higher expressions only on 7 d, but *MYC* and *ERF* showed lower expressions than the *PR* genes. This suggested that SA signaling pathway may be more important in plant resistance to pathogens than JA and ET signaling pathways. We hypothesised that the growth of *Bgt* gradually affected the photosynthesis-related pathway and to some extent the hormone-related pathway.

## Conclusion

In this study, transcriptome sequencing was performed at different stages of *Bgt* infection, and typical genes in signal transduction pathways of different hormones were analyzed. Three hormones, SA, JA, and ET, related pathways were enriched at all periods of *Bgt* infestation. Abscisic acid was also significantly enriched at all stages, indicating that multiple hormones played important roles in wheat defense responses. Expression patterns of most hormone-related genes were as follows: no significant changes in expression trends during 0 h-1 d, an increase in expression trends from 1 d-4 d, and a decrease in expression trends after 4 d. SA, JA, and ET-related pathways were significantly enriched at 1 d-4 d, which therefore could be the main period of hormone action, Some genes in the SA pathway may increase the resistance of wheat to *Bgt* by affecting the expressions of some genes in the JA and ET pathways. Finally, 11 hub genes associated with disease resistance were identified, and these hub genes may be related to the crosstalk of the three hormones, though their specific functions need to be further investigated.

## Materials and methods

### Plant materials and *Bgt* inoculation

The common wheat variety Zhongzuo9504, a susceptible wheat line to *Bgt*, was used in this study. *Bgt* was isolated and purified from the experimental farm of Ningxia University. The above materials were provided by the Crop Genetic Breeding Laboratory of Ningxia University. Zhongzuo 9504 seeds with germination were planted in pots and covered with fresh bags. When the wheat seedlings reached the stage of one leaf and one heart fully unfolding, the first leaf was fixed on a flat plate and inoculated with *Bgt* conidium in the middle leaf section. After the inoculation was completed, the seedlings were moisturized with a cling film cover and placed in the artificial climate incubator (20 ℃ in light for 14 h, 18 ℃ in dark for 10 h). Leaf segments were collected at 0 h, 6 h, 24 h, 48 h, 72 h, 96 h, 120 h, 144 h, and 168 h after inoculation for tissue observation. Leaf segments were sampled at 0 h, 6 h, 1 d, 4 d, and 7 d after inoculation, with three replicates at each time point, and samples were frozen in liquid nitrogen and stored in a -80 °C refrigerator.

### RNA extraction, library construction, and RNA sequencing

A total of 15 collected samples were sent to Genedenovo Biotechnology Co., Ltd (Guangzhou, China) for sequencing on the Illumina sequencing platform. RNA was extracted using Omega Bio-Tek's Omega Plant RNA kit (R6827) and RNA quality was assessed on an Agilent 2100 Bioanalyzer (Agilent Technologies, Palo Alto, CA, USA) and checked using RNase-free agarose gel electrophoresis. After total RNA was extracted, mRNA was enriched by Oligo(dT) beads. Then the enriched mRNA was fragmented into short fragments using fragmentation buffer and reversely transcribed into cDNA by using NEBNext Ultra RNA Library Prep Kit for Illumina (NEB #7530, New England Biolabs, Ipswich, MA, USA). The purified double-stranded cDNA fragments were end repaired, added A base, and ligated to Illumina sequencing adapters. The ligation reaction was purified with the AMPure XP Beads (1.0X). PCR amplification was performed on the screened cDNAs and the PCR products were purified again using AMPure XP beads to obtain the cDNA library. The resulting cDNA library was sequenced using Illumina Novaseq6000 by Gene Denovo Biotechnology Co. (Guangzhou, China).

In order to ensure data quality, the raw reads were quality-controlled using fastp [77] to filter low-quality data and obtain clean reads. Comparative analysis based on reference genomes was performed using HISAT2 software [78]. Since Stringtie can effectively use the abundance of information on transcripts to assemble more transcripts and the assembly results are more accurate, Stringtie was used to assemble transcripts based on the comparison results of HISAT2 [79]. The expression of genes in each sample was calculated using RSEM [80]. The reference genome and genome annotation files were downloaded from the Ensemble Plants (http://plants.ensembl.org/Triticum_aestivum/Info/Index).

### RNA-seq data analysis

The FDR value and log2FC of DEseq2 software [81] were used to screen Differentially Expressed Genes (DEGs), with a default threshold of the adjusted p-value (FDR < 0.05, | log2FC |≥ 1). In order to infer the function of DEGs, Gene Ontology (GO) and Kyoto Encyclopedia of Genes and Genomes (KEGG) enrichment analyses were carried out [82, 83]. The relevant KEGG images in the article have obtained KEGG copyright permission. Trend analysis was performed by Short Time series Expression Miner software [84]. Then, the genes in each trend were analyzed for KEGG/GO functional enrichment, and the *p*-value was calculated through hypothesis testing and adjusted by FDR [85]. KEGG/GO with FDR ≤ 0.05 was defined as a KEGG/GO pathway that is significantly enriched in this trend.

### Multiple plant hormone analysis

Wheat leaves were frozen in liquid nitrogen and ground into a powder (30 Hz, 1 min), and stored at -80 °C. A total of 50 mg of plant samples was placed into a 2 mL plastic microtube and after frozen in liquid nitrogen, dissolved in 1 mL methanol/water/formic acid (15:4:1, V/V/V). 10 μL internal standard mixed solution (100 ng/mL) was added into the extract as internal standards (IS) for the quantitation. The mixture was vortexed for 10 min, then centrifugation for 5 min (12,000 r/min, and 4 °C). The supernatant was transferred to clean plastic microtubes followed by evaporation to dryness and then dissolved in 100 μL 80% methanol (V/V), and filtered through a 0.22 μm membrane filter for LC–MS/MS analysis. Plant hormone contents were determined by MetWare (http://www.metware.cn/) on the AB Sciex QTRAP 6500 LC–MS/MS platform.

### Weighted gene co-expression network analysis

Weighted Gene Co-expression Network Analysis (WGCNA) is an analysis method for analyzing the expression patterns of multiple samples of genes, which clusters genes with similar expression patterns and analyzes the correlation between modules and specific traits or phenotypes [86]. In this study, the 24,209 genes (FPKM $\ge$ 7) were divided into modules and analyzed using WGCNA, and the minimum number of genes per module was set to 50.

### Quantitative real-time PCR and expression validation

In order to verify the results of transcriptome analysis, the relative expression levels of 10 DEGs were detected by qPCR with three biological replicates for each sample. The primers were designed using Primer 3 (https://primer3.org/) and listed in Table 2. The relative expression level was determined using the 2^−ΔΔCt^ method and the wheat actin gene was used as an internal reference gene for qRT-PCR analysis.

**Table 2:** Quantitative real-time PCR Primers

Primer ID	Primer Sequence
TraesCS2A02G215600.1_1_F	ATGGACGACCTTGCTGCC
TraesCS2A02G215600.1_1_R	CACAGGGATTGGGAGCCG
TraesCS2A02G101400.1_1_F	TCTGGAGGTGGAGGAGCC
TraesCS2A02G101400.1_1_R	GGCAGGAGAAGGCGTTGT
TraesCS4A02G005500.1_1_F	TTCTCGTGGCAGGGCATG
TraesCS4A02G005500.1_1_R	CCTCGTCGGCTTCGATCC
TraesCS1A02G301600.1_1_F	TGGAGCCCTGCTTTGACG
TraesCS1A02G301600.1_1_R	CGGGCTGTCGTCTCGTAC
TraesCS5D02G068800.1_1_F	CGCCGTGCTGAAGAGGAA
TraesCS5D02G068800.1_1_R	GGCGAAGTGTCCGAAGCT
TraesCS5D02G244800.1_1_F	CCGGATCTCAGCAGCGAG
TraesCS5D02G244800.1_1_R	GGGAGAGGAAGCCCAGGA
TraesCS5D02G536500.1_1_F	TCCGTGACCCGTTCAAGC
TraesCS5D02G536500.1_1_R	GGAAGTTGCCGAGGCTGT
TraesCS7D02G338800.3_1_F	GGGGTCGGAGGTTTTGGG
TraesCS7D02G338800.3_1_R	CCTTGCTGGGACTTGGGG
TraesCS4A02G116100.2_1_F	TCCGACGCCATGTTCACC
TraesCS4A02G116100.2_1_R	AGCTGCCGGTGTTGAGAC
TraesCS4D02G346000.1_1_F	CCCGTTCAGCAGCCTTCA
TraesCS4D02G346000.1_1_R	GGTGGAACTGCCGAGCTT
actin-F1	TACTCCCTCACAACAACCG
actin-R1	AGAACCTCCACTGAGAACAA

## Data Availability

The wheat line and *Bgt* used in this experiment were provided by the Crop Genetic Breeding Laboratory of Ningxia University. All materials including all relevant raw data described in the manuscript are available upon request. The datasets generated and analyzed during the current study are available in the online repository: https://www.ncbi.nlm.nih.gov/sra/PRJNA1000650, PRJNA1000650.
